# The Relationship of Nitrogen and C/N Ratio with Secondary Metabolites Levels and Antioxidant Activities in Three Varieties of Malaysian Kacip Fatimah (*Labisia pumila* Blume)

**DOI:** 10.3390/molecules16075514

**Published:** 2011-06-29

**Authors:** Mohd Hafiz Ibrahim, Hawa Z.E. Jaafar

**Affiliations:** Department of Crop Science, Faculty of Agriculture, University Putra Malaysia, Serdang 43400, Selangor, Malaysia; Email: mhafizphd@gmail.com (M.H.I.)

**Keywords:** plant medicinal potential, health promoting properties, DPPH, FRAP, total phenolics, total flavonoids

## Abstract

Kacip Fatimah (*Labisia pumila* Blume), one of the most famous and widely used herbs, especially in Southeast Asia, is found to have interesting bioactive compounds and displays health promoting properties. In this study, the antioxidant activities of the methanol extracts of leaves, stems and roots of three varieties of *L. pumila* (var. *alata*, *pumila* and *lanceolata*) were evaluated in an effort to compare and validate the medicinal potential of this indigenous Malaysian herb species. The antioxidant activity determined by the 1,1-diphenyl-2-picrylhydrazyl (DPPH) assay, as well as the total amount of phenolics and flavonoids were the highest in the leaves, followed by the stems and roots in all the varieties. A similar trend was displayed by the ferric reducing antioxidant potential (FRAP) activity, suggesting that the *L. pumila* varieties possess high foliar antioxidant properties. At low FRAP activity concentrations, the values of the leaves’ inhibition activity in the three varieties were significantly higher than those of the stems and roots, with var. *alata* exhibiting higher antioxidant activities and total contents of phenolics and flavonoids compared to the varieties *pumila* and *lanceolata*. The high production of secondary metabolites and antioxidant activities in var. *alata* were firmly related to low nitrogen content and high C/N ratio in plant parts. The study also demonstrated a positive correlation between secondary metabolite content and antioxidant activities, and revealed that the consumption of *L. pumila* could exert several beneficial effects by virtue of its antioxidant activity.

## 1. Introduction

The process of oxidation in the human body damages cell membranes and their structure, including cellular proteins, lipids and DNA, which can cause mutations and ultimately contribute to the development of chronic diseases such as cancer [1,2]. Free radicals are oxygen-based or nitrogen-based molecules with unpaired electrons that are generated by a number of metabolic processes within the body [3]. When food is turned into energy, free radicals are formed by normal oxidation reactions [4]. Many, if not most, of the health benefits of antioxidants arise from their anti-inflammatory properties within the body. Free radical damage is both triggered by inflammation and is itself inflammatory. By lessening free radical damage, antioxidants reduce inflammation [5]. This essential role of antioxidants promotes cardiovascular health [6], inhibits the growth of cancerous tumors and cell masses [7], slows the aging process in the brain and nervous systems, and lessens the risk and severity of neurodegenerative diseases, including Alzheimer’s, Parkinson and Huntington’s diseases [8]. A possible role of antioxidant activity in lessening the severity of asthma has also been observed in a population-based case control study [9].

Antioxidants are substances that delay or inhibit oxidative damage when present in small quantities compared to an oxidizable substrate [10]. Antioxidants affect the process of lipid peroxidation due to the differences in their form of action. Hence, antioxidants can help in disease prevention by effectively neutralizing free radicals or the inhibiting the damages they create [11]. According to Rice *et al.* [12] the number of hydroxyl groups and the amount and types of conjugation are the two most important factors in the antioxidative potential of phenolic compounds. Many common antioxidant compounds are found in fruits, vegetables and spices/herbs [13]. Antioxidant activities have been reported in several plants, including *Ocimum sanctum*, *Piper cubeba*, *Allium sativum*, *Terminalia bellerica*, *Camellia sinensis*, *Zingiber officinale* and several Indian and Chinese herbs. The majority of the antioxidative activity could be due to the flavones, isoflavones, flavonoids, antocyanin, coumarins lignans, catechin and isocatechins contents found in these plants [14].

In addition to their role as health supplements, antioxidants are also added into food preparations to hinder oxidation, usually initiated by free radicals formed during the exposure of food to environmental factors such as air, temperature and light [15]. Ascorbic acid, benzoic acid, carotenoids, cinnamic acids, folic acid, tocopherols, tocotrienols, *etc*., are among the antioxidants produced by plants for their own use. Among them, β-carotene, ascorbic acid and α-tocopherol are extensively used as antioxidants in the pharmaceutical and food industries [16]. Synthetic antioxidants such as butylated hydroxytoluene (BHT) and butylated hydroxyanisole (BHA) have been widely used for many years to retard lipid oxidation, but the safety of using these synthetic antioxidants in the food industry has become a concern among scientists, leading to current increased interest in uncovering natural antioxidants [17].

The natural antioxidants cover a broad range of compounds including phenolics, flavonoids, nitrogen compounds and caretenoids [18]. As a result, many plant species have been evaluated for their antioxidative properties in pharmacology and in food preparations. Among these plants species, *Labisia pumila* Blume (Myrsinacea family), locally known as Kacip Fatimah in Malaysia, has received a lot of attention among scientists, herbalists and the herbal industry. This is because *L. pumila* is a popular herb that has long been recognized by the traditional practitioner to possess therapeutic properties and high levels of phenolic and flavonoid compounds [19,20]. These polyphenolic compounds have received considerable attention because of their protective role against cancer and heart disease, attributed to their antioxidative activity against reactive oxygen species, which was reported to be higher than that of vitamins C and E [21]. Both phenolic acids and flavonoids are believed to be responsible for the wide spectrum of pharmacological activities, and hence, could be contributing towards this herb’s medicinal potentiality [22]. *L. pumila* has been used as medicinal treatments for dysentry, flatulance, dysmonorrhea and gonorrhoea [23]; however, documentation of its phytochemical properties and profiling is still lacking and needs immediate attention in order to expedite the development of the Malaysian herbal industry. Domestically, there are three recognized varieties of *L. pumila* namely *L. pumila* var. *alata*, *L. pumila* var. *pumila* and *L. pumila* var. *lanceolata* [24]. Each variety commands different uses [20].

Previous research by Ibrahim *et al.* [25] had shown that a high production of secondary metabolite compounds was elicited in *L. pumila* by a high C/N ratio and low nitrogen fertilization, especially when plants were exposed to elevated CO_2_ levels. Under nutrient resource limitation, especially of nitrogen, which restricts growth to a greater extent than photosynthesis, plants usually show an increase in the C/N ratio and this signifies an increased production of secondary metabolites [26]. It was also reported that the increase in C/N ratio in plants might be due to low nitrogen absorption or fertilization of the plants [27]. Lindroth [28] proposed that the C/N ratio was a good indicator of increased production of secondary metabolites and low levels of plant nitrogen. Previous studies on the antioxidative properties of *L. pumila* were limited to the varieties *alata* and *pumila,* and activities confined only to the leaves (Norhaiza *et al.* [29]). There are no reports on the antioxidative differences among the three varieties due to varied nitrogen levels and the relationship with the C/N ratio. Therefore, a study was carried out to determine the antioxidant activity and secondary metabolites (total phenolics and flavonoids) of methanolic extracts and the relationships among secondary metabolites, antioxidant activity, nitrogen (N) and carbon to nitrogen ratio (C/N) of three varieties of *L. pumila*, namely *L. pumila* var. *alata*, *L .pumila* var. *pumila* and *L. pumila* var. *lanceolata*.

## 2. Results and Discussion

### 2.1. Total Phenolics and Total Flavonoids Profiling

Total phenolics and flavonoids in *L. pumila* were influenced by the interaction between varieties and plant parts (P ≤ 0.01). Total phenolics content was significantly higher in the leaf of var. *alata* than the leaf of var. *pumila* and var. *lanceolata* by approximately 5% and 18%, respectively; followed by the stemd of var. *alata, pumila* and *lanceolata*, that recorded 0.98, 0.87 and 0.85 mg gallic acid/g dry weight, respectively (Table 1). However, among the varieties, there was no statistical significance observed in the stem profiles. The lowest total phenolics were recorded in the roots, particularly in var. *lanceolata* (0.67 mg gallic acid/g dry weight) followed by var. *alata* and var. *pumila* with corresponding values of 0.87 and 0.76 mg gallic acid/g dry weight. A similar trend to the total phenolics one was observed for total flavonoids, where the highest value was registered in the leaves of var. *alata* (0.91 mg rutin/g dry weight) and the lowest in the roots of var. *lanceolata* (0.23 mg rutin/g dry weight).

These results appear to contradict the findings of Norhaiza *et al.* [29], who reported higher total flavonoids (rutin) in the leaves of var. *pumila* compared to var. *alata*, with no significant differences between total phenolics in the two varieties. In the current work *L. pumila* demonstrated the highest values of total phenolics (gallic acid) and flavonoids (rutin) in var. *alata* compared to the other two varieties. The variability in the results might be due to the differences in ages of the plants used for sampling, as Norhaiza *et al.* [29] had used a 12-month old plant whilst we used a 4-month old seedling. Our result, on the other hand, was in agreement with the data presented by Karimi *et al*. [30], who demonstrated the highest contents of total phenolics and flavonoids in the leaves, followed by the stems and then the roots in all the three varieties of *L. pumila*, with var. *alata* exhibiting higher values than the others. When the compositions of phenolics and flavonoids were analyzed by high performance liquid chromatography (HPLC), gallic acid was found to be the main phenolic in all three varieties. They also found that pyrogallol and kaempherol were the main flavonoid constituents in var. *alata.* However, the main flavonoids in var. *pumila* were rutin and quercetin, and in var. *lanceolata*, it was kaempferol. It could be concluded that the main flavonoid constituents of the three varieties of *L. pumila* were pyragallol, kaempherol, rutin and quercetin; and together with gallic acid, the three varieties were shown to possess high anticancer activities [30]. Ferry *et al.* [31] and Ranelletti *et al*. [32] have also shown that these phenolics and flavonoids had anticancer activities, and that they were able to inhibit cancer cell growth. Gallic acid and rutin were reported to have high scavenging activity and act as treatments for diabetes, albuminaria, psoriasis and external haemorrhoids [33,34]. Some studies have reported that gallic acid, rutin, pyragallol, kaempherol and quercetin play an important role in the prevention of cancer [35]. The present results suggest that the three varieties of *L. pumila* have potential antimicrobial and anticancer activities.

**Table 1:** Total phenolics and flavonoids contents in different parts of three varieties of *L. pumila*.

Varieties	Plant parts	Total phenolics ^1^	Total flavonoids ^2^
	Leaves	1.64 ± 0.03 ^a,^*	0.91 ± 0.10 ^a,^*
**Alata**	Stems	0.98 ± 0.02 ^b^	0.54 ± 0.05 ^b^
	Roots	0.87 ± 0.02 ^c^	0.34 ± 0.01 ^c^
	Leaves	1.56 ± 0.02 ^a^	0.87 ± 0.01 ^a^
**Pumila**	Stems	0.87 ± 0.04 ^b^	0.31 ± 0.07 ^b^
	Roots	0.76 ± 0.03 ^c^	0.25 ± 0.05 ^c^
	Leaves	1.34 ± 0.12 ^a^	0.79 ± 0.02 ^a^
**Lanceolata**	Stems	0.85 ± 0.02 ^b^	0.24 ± 0.0 ^b^
	Roots	0.67 ± 0.01 ^c^	0.23 ± 0.03 ^c^

All analyses are mean ± standard error of mean (SEM), N = 27; *: Similar letters denote insignificant differences at P ≤ 0.05 among the means; ^1^: Expressed as mg gallic acid/g dry weight; ^2^: Expressed as mg rutin/g dry weight.

### 2.2. Radical Scavenging Activity

#### 2.2.1. 1,1-Diphenyl-2-picrylhydrazyl (DPPH) assay

**Figure 1:**
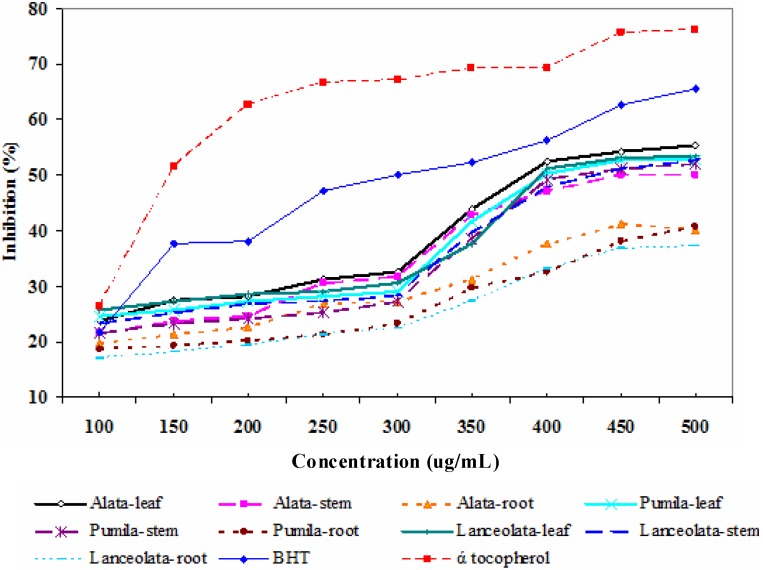
DPPH radical scavenging activity in different parts of three varieties of *L. pumila* compared to the positive controls, BHT and α-tocopherol.

It was observed from the results that methanolic extract of leaves and stem of *L. pumila* had higher activity than the root part (Figure 1). At a concentration of 400 µg/mL the scavenging activity of the methanolic extract of var. *alata* leaves reached 51.3%, whereas at the same concentration the activities recorded in stem and the root were 47.2% and 37.8%, respectively. Using the same concentration (400 µg/mL) the scavenging activities in the leaves, stem and root of var. *pumila* registered 50.23%, 49.2% and 32.7%, respectively. Meanwhile in var. *lanceolata* the DPPH inhibition at 400 µg/mL in the leaves, stems and roots was 51.2%, 47.8% and 33.2%, respectively. The enhanced DPPH activity in the leaves of *L. pumila* might due to higher levels of polyphenolic compounds that contribute to the higher antioxidant activity [36]. The enhanced antioxidant activity is believed mainly due to their redox properties that plays an important role in absorbing and neutralizing free radicals, quenching singlet and triplet oxygen or decomposition of peroxides [37]. In the present study, the effect of antioxidant on DPPH might be due to their hydrogen donating ability [38,39].

The results of the current work also show that the DPPH radical scavenging abilities of the plant extracts at 400 µg/mL were less than those of the reference antioxidants butylated hydroxytoluene (BHT, 58.2%) and α-tocopherol (69.27%). This study showed that *L. pumila* methanolic extract has high free radical scavenging activity and, hence, it could be used as a radical scavenger, acting possibly as the primary antioxidant. The antioxidant activities of the leaves were highest (52.83%–55.32%), followed by the stems (50.14%–52.72%) and the least in the roots (37.2%–40.73%). Based on the results, it is possible that several compounds of different polarities may contribute to the antioxidative properties of *L. pumila* leaves, stems and roots extracts. In addition, antioxidant activities observed in *L. pumila* varieties might be due to synergistic effects of more than two components that may be present in the plant [40].

### 2.3. Reducing Ability

#### 2.3.1. Ferric reducing antioxidant potential (FRAP)

The ferric reducing antioxidant potential (FRAP) was influenced by the interaction effects between varieties and plant parts (P ≤ 0.01). The FRAP activity was found to be highest in the leaves, followed by the stems and roots, and highest in var. *alata*, followed by var. *pumila* and var. *lanceolata*. The reducing ability of different parts of *L. pumila* extracts was in the range of 401.23–680.68 µm of Fe (II) dry weight (Table 2). The result indicated that *L. pumila* leaves have high ability to reduce ferric ions than stem and root parts [29]. In the leaves, stems and roots, the antioxidant potentiality of three varieties of *L. pumila* was estimated from their ability to reduce 2,4,6-tripyridyl-*s*-triazine (TPTZ)-Fe (III) complex to TPTZ-Fe (II). The FRAP values for the methanolic extracts of the leaves, stems and roots in all varieties were statistically significantly lower than those of vitamin C and α-tocopherol, but higher than that of BHT.

**Table 2:** Total antioxidant (FRAP) activity in different parts of three varieties of *L pumila*. BHT, α-tocopherol and vitamin c were used as positive controls.

Varieties	Extract source	FRAP ^1^
	Leaves	680.68 ± 34.51 ^c,^*
**Alata**	Stems	537.65 ± 33.23 ^e^
	Roots	435.23 ± 12.67 ^f^
	Leaves	600.45 ± 16.90 ^d^
**Pumila**	Stems	512.56 ± 12.89 ^e^
	Roots	401.23 ± 12.89 ^f^
	Leaves	599.87 ± 12.83 ^d^
**Lanceolata**	Stems	425.67 ± 10.45 ^e^
	Roots	421.61 ± 12.34 ^f^
**Controls**	BHT	84.32 ± 56.34 ^g^
	α-tocopherol	953.01 ± 45.67 ^b^
	Vitamin C	3301.25 ± 34.56 ^a^

All analyses are mean ± standard error of mean (SEM), N = 27; *: Similar letters denote insignificant differences at P ≤ 0.05 among the means; ^1^: Results expressed in percent of free radical inhibition.

The ferric reducing ability (FRAP assay) is widely used in the evaluation of the antioxidant strength of dietary polyphenols [41]. The antioxidant activity is found to be linearly proportional to the phenolic and flavonoid content [42]. Yen *et al.* [43] reported that the ferric reducing power of bioactive compounds was associated with antioxidant activity. Gill *et al.* [44] have reported a strong positive relationship between total phenolic compounds and antioxidant activity, which appears to be a similar trend to that shown by the present study, where total phenolics and flavonoids have significant positive relationships (P ≤ 0.01) with FRAP activity, exhibiting values of R^2^ = 0.87 and R^2^ = 0.75, respectively (Table 3). This result implies that the high DPPH and FRAP values of *L. pumila* might be due to high accumulations of total phenolics and flavonoids in the plants.

**Table 3:** The correlationship among the parameters recorded in the study (total phenolics (TP), total flavonoids (TF), DPPH, FRAP, C, N and C/N).

	1	2	3	4	5	6	7
**1.TP**	1.00						
**2.TF**	0.90 *	1.00					
**3. DPPH**	0.89 *	0.78 *	1.00				
**4. FRAP**	0.87 *	0.75 **	0.83 *	1.00			
**5. C**	0.32	0.02	0.21	0.09	1.00		
**6. N**	−0.87 *	−0.87 **	−0.71 *	−0.67*	0.42	1.00	
**7. C/N**	0.71 *	0.70 *	−0.72 *	−0.69*	0.32	−0.54 *	1.00

* and ** are statistically significant at 5% and 1%.

### 2.4. Total Carbon, Nitrogen and C/N Profiling

The nitrogen and C/N ratio were influenced by the interaction between varieties and plant parts (P ≤ 0.05). However, there were no significant differences in total carbon among varieties and plant parts, as shown in Table 4 (P ≥ 0.05).

**Table 4:** Total carbon, nitrogen and C/N in different parts of three varieties of *L. pumila*.

Varieties	Plant Parts	Carbon (%)	Nitrogen (%)	C/N
	Leaves	37.61 ± 0.21 ^a,^*	1.15 ± 0.45 ^c,^*	31.94 ± 0.34 ^a,^*
**Alata**	Stems	38.22 ± 0.34 ^a^	1.87 ± 0.01 ^b^	20.44 ± 0.45 ^c^
	Roots	37.32 ± 0.21 ^a^	1.95 ± 0.02 ^a^	19.24 ± 1.21 ^d^
	Leaves	38.74 ± 0.32 ^a^	1.31 ± 0.32 ^c^	29.36 ± 0.23 ^a^
**Pumila**	Stems	37.21 ± 0.32 ^a^	1.72 ± 0.21 ^b^	21.19 ± 2.12 ^c^
	Roots	36.87 ± 0.42 ^a^	1.85 ± 0.23 ^a^	20.43 ± 0.23 ^d^
	Leaves	38.13 ± 0.43 ^a^	1.55 ± 0.01 ^c^	25.31 ± 0.43 ^b^
**Lanceolata**	Stems	36.52 ± 0.10 ^a^	1.73 ± 0.02 ^b^	21.46 ± 0.32 ^c^
	Roots	37.21 ± 0.13 ^a^	1.93 ± 0.43 ^a^	19.05 ± 0.01 ^d^

All analyses are mean ± standard error of mean (SEM), N = 27; Means not sharing a common single letter were significantly different at P ≤ 0.05.

The highest plant nitrogen was recorded in var. *alata* root (1.95% N), followed by var. *lanceolata* root (1.93% N), var. *pumila* root (1.85% N), var. *alata* stem (1.87% N), var. *lanceolata s*tem (1.73% N), var. *pumila* stem (1.72% N), var. *lanceolata* leaf (1.55% N), var. *pumila* leaf (1.31% N) and var. *alata* leaf (1.15% N). Meanwhile, the inverse trend was observed in C/N ratio, where the highest C/N ratio was shown by the leaves followed by the stems and the lowest in the roots of all the varieties, with the highest value being registered in var. *alata* leaf (31.94) and the lowest in var. *lanceolata* root (19.05). However, there was no significance difference observed among roots of all varieties. The differences in accumulation of secondary metabolites and antioxidant activity in different varieties and plant parts might be due to variabilities in N and C/N ratio. The highest accumulation of plant secondary metabolites and antioxidant activity was found to be in the varieties and plant parts that had low N content, which was the leaf of var. *alata*, while the lowest accumulation and antioxidant activity was in var. *lanceolata* root which had a high N content. At the same time, var. *alata* leaf and var. *lanceolata* root had the highest and lowest C/N ratios, respectively. These findings were in agreement with a previous study by Ibrahim *et al.* [25], who found that *L. pumila* which displayed the lowest N and high C/N was found to accumulate higher total phenolics and flavonoids.

From the correlations in Table 3, it was found that N had a significant negative relationship with total phenolics (R^2^ = −0.87; P ≤ 0.05), total flavonoids (R^2^ = −0.87; P ≤ 0.01), DPPH (R^2^ = −0.71; P ≤ 0.05), FRAP (R^2^ = −0.67; P ≤ 0.05) and C/N (R^2^ = −0.54; P ≤ 0.05). Results indicate that secondary metabolites, antioxidant activity and C/N ratio might be up-regulated under low nitrogen conditions. The increase in secondary metabolites, antioxidant activity and C/N ratio under N limitation has been well documented [45,46,47]. In the current study, the differences in varieties and plant parts in the accumulation of secondary metabolites and antioxidant activity might be due to differences in the absorption rates of plant N in different varieties and plant parts [48]. In the present study, we have identified high C/N ratio as a good indicator of the production of secondary metabolites and antioxidant activity in *L. pumila*. Further studies are needed to identify the phenolic and flavonoid components that contributed to the high antioxidant activities in leaves of var. *alata*.

## 3. Experimental

### 3.1. Plant Material and Maintenance

The experiments were carried out under a growth house at Ladang 2, Faculty of Agriculture Glasshouse Complex, Universiti Putra Malaysia (longitude 101° 44’ N and latitude 2° 58’S, 68 m above sea level) with a mean atmospheric pressure of 1.013 kPa. Three-month old *L. pumila* seedlings of var. *alata*, var. *pumila* and var. *lanceolata* were left for a month to acclimatize in a nursery until ready for the treatments. The soilless media used for the plants was comprised of burnt rice husks, coco peat and chicken dung at a ratio of 5:5.1. The plants were grown under glasshouse conditions under a daily irradiance of approximately 300 µmol·m^−^2·s^−1^. The experiment was designed as a completely randomized CRD factorial design replicated three times, whereby three varieties of *L. pumila* and three plant parts were used as factors. Healthy and uniform seedlings in terms of leaf numbers were selected from the three varieties. The plants were harvested after 15 weeks of acclimation, with the leaves, stems, and roots being separated. Once dried, they were all kept at −80 °C for future analysis.

### 3.2. Extract Preparation

Leaves, stems and root were freeze dried to constant weights prior to being used in the extraction. For antioxidant analysis, the leaves, stems, and roots were powdered and 1 gram of the powder was extracted continuously with methanol (50 mL). The solution was then swirled for 1 h at room temperature using an orbital shaker. Extracts were then filtered under suction and stored at −20 °C for further use.

### 3.3. Quantification of Total Phenolics and Total Flavonoids

The method of extraction and quantification of total phenolics and flavonoids followed after Jaafar *et al.* [49]. An amount of ground tissue sample (0.1 g) was extracted with 80% ethanol (10 mL) on an orbital shaker for 120 min at 50 °C. The mixture was subsequently filtered (Whatman™ No.1), and the filtrate was used for the quantification of total phenolics and total flavonoids. Folin-Ciocalteau reagent (diluted 10-fold) was used to determine the total phenolics content of the leaf samples. Two hundred µL of the sample extract was mixed with Folin-Ciocalteau reagent (1.5 mL) and allowed to stand at 22 °C for 5 min before adding NaNO_3_ solution (1.5 mL, 60 g L^−1^). After two hours at 22 °C, absorbance was measured at 725 nm. The results were expressed as mg g^−1^ gallic acid equivalent (mg GAE/g dry sample). For total flavonoids determination, sample (1 mL) was mixed with NaNO_3_ (0.3 mL) in a test tube covered with aluminium foil, and left for 5 min. Then 10% AlCl_3_ (0.3 mL) was added, followed by addition of 1 M NaOH (2 mL) and the absorbance was measured at 510 nm using rutin as a standard (results are expressed as mg rutin/g dry sample).

### 3.4. Determination of Antioxidant Activities

#### 3.4.1. DPPH radical scavenging assay

The DPPH free radical scavenging activity of each sample was determined according to the method described by Wong *et al.* [36]. A solution of 0.1 mM DPPH in methanol was prepared. The initial absorbance of the DPPH in methanol was measured at 515 nm. An aliquot (40 µL) of an extract was added to methanolic DPPH solution (3 mL). The change in absorbance at 515 nm was measured after 30 min. The antiradical activity (AA) was determined using the following formula:

AA% = 100 − [(Abs:sample − Abs:empty sample)]* 100) / Abs:control

The optic density of the samples, the control and the empty samples were measured in comparison with methanol. One synthetic antioxidant, BHT (butylated hydroxytoluene), and α-tocopherol, were used as positive controls. 

#### 3.4.2. Reducing ability (FRAP assay)

The ability to reduce ferric ions was measured using modifying methods of Benzie and Strain [50]. An aliquot (200 µL) of the extract with appropriate dilution was added to FRAP reagent (3 mL, 10 parts of 300 mM sodium acetate buffer at pH 3.6, 1 part of 10 mM TPTZ solution and 1 part of 20 mM FeCl_3_ 6H_2_O solution) and the reaction mixture was incubated in a water bath at 37 °C. The increase in absorbance at 593 nm was measured after 30 min. The antioxidant capacity based on the ability to reduce ferric ions of the extract was expressed as in µM Fe (II)/g dry mass and compared with those of standards for BHT, ascorbic acid, and α-tocopherol.

### 3.5. Total Carbon, Nitrogen and C:N Ratio

Total carbon and C/N ratio were measured by using a CNS 2000 analyzer (Model A Analyst 300, LECO Inc, USA). This was performed by placing 0.05 g of ground leaf sample into the combustion boat. Successively, the combustion boat was transferred to the loader before the sample was burned at 1,350 °C to obtain the reading of total carbon and nitrogen content of the samples [51].

### 3.6. Statistical Analysis

Data were analyzed using analysis of variance by SAS version 17. Mean separation test between treatments was performed using the Duncan multiple range test and standard error of differences between means was calculated with the assumption that data were normally distributed and equally replicated.

## 4. Conclusions

*L. pumila* was found to have a high antioxidative activity and among the three varieties examined, var. *alata* seemed to possess good medicinal potential. The high levels of total phenolics and flavonoids in var. *alata* indicated high antioxidant activities. The high secondary metabolites in var. *alata* might be attributed to low N and high C/N ratio that favor the accumulation of secondary metabolites, hence increasing the antioxidative potential of the plant. Further work is needed in the future to establish the components that could have contributed to the high antioxidant activities observed in the present study.
